# Association of Sleep Duration With All- and Major-Cause Mortality Among Adults in Japan, China, Singapore, and Korea

**DOI:** 10.1001/jamanetworkopen.2021.22837

**Published:** 2021-09-03

**Authors:** Thomas Svensson, Eiko Saito, Akiko Kishi Svensson, Olle Melander, Marju Orho-Melander, Masaru Mimura, Shafiur Rahman, Norie Sawada, Woon-Puay Koh, Xiao-Ou Shu, Ichiro Tsuji, Seiki Kanemura, Sue K. Park, Chisato Nagata, Shoichiro Tsugane, Hui Cai, Jian-Min Yuan, Sanae Matsuyama, Yumi Sugawara, Keiko Wada, Keun-Young Yoo, Kee Seng Chia, Paolo Boffetta, Habibul Ahsan, Wei Zheng, Daehee Kang, John D. Potter, Manami Inoue

**Affiliations:** 1 Epidemiology and Prevention Group, Center for Public Health Sciences, National Cancer Center, Tsukiji, Chuo-ku, Tokyo, Japan; 2 Precision Health, Department of Bioengineering, Graduate School of Engineering, The University of Tokyo, Bunkyo-ku, Tokyo, Japan; 3 Department of Clinical Sciences, Lund University, Skåne University Hospital, Malmö, Sweden; 4 Kanagawa University of Human Services School of Health Innovation, Kawasaki-ku, Kawasaki-shi, Kanagawa, Japan; 5 Department of Neuropsychiatry, Keio University School of Medicine, Shinjuku-ku, Tokyo, Japan; 6 Center for Cancer Control and Information Services, Division of Cancer Statistics Integration, National Cancer Center, Tokyo, Japan; 7 Department of Diabetes and Metabolic Diseases, University of Tokyo, Hongo, Bunkyo-ku, Tokyo, Japan; 8 Department of Internal Medicine, Skåne University Hospital, Malmö, Sweden; 9 Center for Public Health Sciences, Division of Prevention, National Cancer Center, Tokyo, Japan; 10 Research Center for Child Mental Development, Hamamatsu University School of Medicine, Hamamatsu, Japan; 11 Healthy Longevity Translational Research Programme, Yong Loo Lin School of Medicine, National University of Singapore, Singapore; 12 Vanderbilt-Ingram Cancer Center, Vanderbilt Epidemiology Center, Division of Epidemiology, Vanderbilt University Medical Center, Nashville, Tennessee; 13 Tohoku University Graduate School of Medicine, Sendai, Miyagi, Japan; 14 Department of Preventive Medicine, Seoul National University College of Medicine, Seoul, Republic of Korea; 15 Department of Epidemiology and Preventive Medicine, Gifu University Graduate School of Medicine, Gifu, Japan; 16 UPMC Hillman Cancer Center, Division of Cancer Control and Population Sciences, University of Pittsburgh, Pittsburgh, Pennsylvania; 17 Department of Epidemiology, Graduate School of Public Health, University of Pittsburgh, Pittsburgh, Pennsylvania; 18 Seoul National University College of Medicine, Seoul, South Korea; 19 National University of Singapore, Singapore; 20 Stony Brook Cancer Center, Stony Brook University, Stony Brook, New York; 21 Department of Medical and Surgical Sciences, University of Bologna, Bologna, Italy; 22 Department of Public Health Sciences, The University of Chicago, Chicago, Illinois; 23 Seoul National University College of Medicine, Seoul, South Korea; 24 Research Centre for Hauora and Health, Massey University, Wellington, New Zealand

## Abstract

**Importance:**

The association between long sleep duration and mortality appears stronger in East Asian populations than in North American or European populations.

**Objectives:**

To assess the sex-specific association between sleep duration and all-cause and major-cause mortality in a pooled longitudinal cohort and to stratify the association by age and body mass index.

**Design, Setting, and Participants:**

This cohort study of individual-level data from 9 cohorts in the Asia Cohort Consortium was performed from January 1, 1984, to December 31, 2002. The final population included participants from Japan, China, Singapore, and Korea. Mean (SD) follow-up time was 14.0 (5.0) years for men and 13.4 (5.3) years for women. Data analysis was performed from August 1, 2018, to May 31, 2021.

**Exposures:**

Self-reported sleep duration, with 7 hours as the reference category.

**Main Outcomes and Measures:**

Mortality, including deaths from all causes, cardiovascular disease, cancer, and other causes. Sex-specific hazard ratios (HRs) and 95% CIs were estimated using Cox proportional hazards regression with shared frailty models adjusted for age and the key self-reported covariates of marital status, body mass index, smoking status, alcohol consumption, physical activity, history of diabetes and hypertension, and menopausal status (for women).

**Results:**

For 322 721 participants (mean [SD] age, 54.5 [9.2] years; 178 542 [55.3%] female), 19 419 deaths occurred among men (mean [SD] age of men, 53.6 [9.0] years) and 13 768 deaths among women (mean [SD] age of women, 55.3 [9.2] years). A sleep duration of 7 hours was the nadir for associations with all-cause, cardiovascular disease, and other-cause mortality in both men and women, whereas 8 hours was the mode sleep duration among men and the second most common sleep duration among women. The association between sleep duration and all-cause mortality was J-shaped for both men and women. The greatest association for all-cause mortality was with sleep durations of 10 hours or longer for both men (hazard ratio [HR], 1.34; 95% CI, 1.26-1.44) and women (HR, 1.48; 95% CI, 1.36-1.61). Sex was a significant modifier of the association between sleep duration and mortality from cardiovascular disease (χ^2^_5_ = 13.47, *P* = .02), cancer (χ^2^_5_ = 16.04, *P* = .007), and other causes (χ^2^_5_ = 12.79, *P* = .03). Age was a significant modifier of the associations among men only (all-cause mortality: χ^2^_5_ = 41.49, *P* < .001; cancer: χ^2^_5_ = 27.94, *P* < .001; other-cause mortality: χ^2^_5_ = 24.51, *P* < .001).

**Conclusions and Relevance:**

The findings of this cohort study suggest that sleep duration is a behavioral risk factor for mortality in both men and women. Age was a modifier of the association between sleep duration in men but not in women. Sleep duration recommendations in these populations may need to be considered in the context of sex and age.

## Key Points

**Question:**

What is the association between sleep duration and mortality outcomes in individuals from East Asia?

**Findings:**

In this cohort study of 322 721 adult participants, when compared with 7 hours, all other sleep durations were associated with a significantly increased mortality risk. Age and sex were significant modifiers of the associations between sleep duration and death from all causes (age), cardiovascular disease (sex), cancer (age and sex), and other causes (age and sex), and age was a modifier of the association only among men.

**Meaning:**

These findings suggest that sleep duration recommendations for East Asian populations may need to be considered in the context of sex and age.

## Teaser

This cohort study examines whether sleep duration is associated with all-cause and major-cause mortality in individuals from Japan, China, Singapore, and Korea.

## Introduction

Short and long sleep durations are emerging as important behavioral risk factors for mortality. Meta-analyses^1,2,3,4,5,6^ of prospective studies indicate that both short and long sleep durations are associated with all-cause mortality. Despite the strong evidence from meta-analyses, there are a number of limitations to consider^7^: the individual studies included in meta-analyses differ in their follow-up times and selection of confounders and apply heterogenous definitions of what constitutes short and long sleep durations. In addition, the association between long sleep duration and mortality appears stronger in East Asian populations than in North American or European populations.^1^

Moreover, the association between sleep duration and mortality may differ among population subgroups; individual studies have already highlighted mortality risks with sleep duration that vary by sex^6,8^ and age.^6,8,9^ However, in East Asian populations, body mass index (BMI; calculated as weight in kilograms divided by height in meters squared) is associated with sleep duration^10^ as well as with cardiovascular disease (CVD) and cancer mortality^11^ and should therefore also be considered as a potential confounder or modifier of the association between sleep duration and mortality outcomes. To the best of our knowledge, no study has stratified analyses according to BMI in a predominantly healthy population.

The primary objective of this study was to conduct the largest pooled individual-level analysis to date of the sex-specific association between sleep duration and all-cause and major-cause mortality in an overall healthy East Asian population. The secondary and tertiary objectives were to conduct sex-specific analyses that were additionally stratified by age and BMI. We hypothesized that there would be an overall U-shaped association between sleep duration and mortality for both men and women but that associations would differ when including additional variables.

## Methods

Details of the Asia Cohort Consortium (ACC) have been described elsewhere.^11^ In brief, the ACC is a consortium of more than 30 cohorts from Asia: Bangladesh, China, India, Iran, Japan, Korea, Malaysia, Mongolia, Singapore, and Taiwan. The ACC was established to allow for sufficient sample size and power to investigate the association among environmental exposures, genetics, including their interactions, and the cause and mortality of diseases. The inclusion of cohorts in the current study was based on the availability of information on sleep duration. The study thus included 9 cohorts from 4 countries (5 cohorts from Japan, 2 from China, 1 from Singapore, and 1 from Korea; hereafter referred to as East Asian), with a total of 450 532 participants. The cohorts were studied from January 1, 1984, to December 31, 2002. The individual participant data provided by each cohort were pooled and harmonized centrally (eMethods in the Supplement). Data analysis was performed from August 1, 2018, to May 31, 2021. Pooled analysis of the ACC cohorts was approved by the ethical committee of the National Cancer Center Japan, and each study was approved by respective ethics committees overseeing the participating studies. All study participants provided written informed consent to the respective cohort studies and this analysis. All data were deidentified. The study followed the Strengthening the Reporting of Observational Studies in Epidemiology (STROBE) reporting guideline.

### Exclusion of Study Participants

Of the 450 532 eligible study participants, we excluded those with missing information on sex (n = 114) and follow-up time (n = 6116), those whose follow-up time was negative, 0, or of an unreasonable value (n = 58), and those younger than 18 years (n = 522) (eFigure in the Supplement). We further excluded participants who did not provide any information on sleep duration (n = 30 445) and for whom continuous sleep duration was an outlier value of more than 3 interquartile ranges below or above the first and fourth quartiles (n = 1477). Participants with missing information on confounders (n = 52 481), with a self-reported history of cancer or CVD at baseline (n = 26 490), and those who died in the first 5 years of follow-up (n = 10 108) were also excluded. The final study population included 322 721 participants.

### Main Exposure

The main exposure in the current study was self-reported sleep duration (eMethods in the Supplement). For all but 1 of the participating cohorts (Shanghai Women’s Health Study), sleep duration was assessed at the time of the baseline survey. For the Shanghai Women’s Health Study, sleep duration was assessed at the third follow-up survey. After exclusion of outlier values (as detailed above), the continuous variable was categorized into 6 groups: 5 hours or less, 6 hours, 7 hours, 8 hours, 9 hours, and 10 hours or more. Seven hours of sleep duration was chosen as the reference category to facilitate comparison of results with other studies, most of which use 7 hours of sleep as the reference.

### Outcome Ascertainment and Follow-up

Mortality outcomes were ascertained using death certificates, with causes of death defined according to the *International Classification of Diseases, Ninth Revision (ICD-9)* and *International Statistical Classification of Diseases and Related Health Problems, Tenth Revision (ICD-10)*. The outcomes of the current study were mortality, comprising death from all causes, CVD, cancer, and other causes. Details of the outcome definitions are given in the eMethods in the Supplement.

Follow-up time was calculated from the time of providing information on sleep duration to the date of death or the end of the follow-up period, whichever came first. Participants lost to follow-up were censored at their last known date of inclusion in the study.

### Statistical Analysis

Comparison of baseline characteristics among sleep duration categories was performed using the χ^2^ test and 1-way analysis of variance for categorical and continuous variables, respectively. Sex-specific hazard ratios (HRs) and 95% CIs were estimated using Cox proportional hazards regression with shared frailty models. Shared frailty models account for possible unobserved between-study heterogeneity^12^ with the random effects defined at the cohort level.

Minimally adjusted models (model 1) were adjusted for age at start of follow-up, study area, and marital status. The multivariable models (model 2) were additionally adjusted for BMI, smoking status, alcohol consumption, physical activity, history of diabetes and hypertension, and, for women, menopausal status.

Prespecified stratified analyses were conducted according to age (<65 years or ≥65 years) and BMI (<25 or ≥25). Modification of the association was determined using the likelihood ratio test; the multivariable models, including an interaction term between sleep duration and the stratifying variable, were compared to models without this term.

All statistical analyses were performed using Stata software, version 14.2 SE (StataCorp LLC). A 2-tailed *P* < .05 was considered to be statistically significant.

## Results

A total of 322 721 individuals (mean [SD] age, 54.5 [9.2] years; 178 542 [55.3%] female) participated in the study. The baseline characteristics of the male cohort (mean [SD] age of men, 53.6 [9.0] years) are given in Table 1 and of the female cohort (mean [SD] age of women, 55.3 [9.2] years) in Table 2. The total follow-up time was 2 022 289 person-years for men and 2 392 037 person-years for women, corresponding to a mean (SD) follow-up time of 14.0 (5.0) years for men and 13.4 (5.3) years for women. The mode sleep duration (n = 50 570 [35.1%]) among the 144 179 men included in the pooled analyses was 8 hours, whereas the mode sleep duration (n = 60 319 [33.8%]) among the 178 542 women was 7 hours. Details of cohort-specific sleep durations are available in eTable 1 in the Supplement.

**Table 1.** Baseline Characteristics for 144 179 East Asian Men According to Sleep Duration^a^

Characteristic	Sleep duration, h						*P* value^b^
	≤5	6	7	8	9	≥10	
No. of individuals^c^	7797 (5.4)	25 860 (17.9)	45 768 (31.7)	50 570 (35.1)	9180 (6.4)	5004 (3.5)	NA
Age, mean (SD), y	55.1 (9.2)	53.5 (8.9)	52.6 (8.7)	53.6 (8.9)	56.1 (9.4)	57.3 (9.8)	<.001
Married	7072 (90.7)	23 792 (92.0)	42 058 (91.9)	46 220 (91.4)	8375 (91.2)	4455 (89.0)	<.001
Smoking status							
Never	2412 (30.9)	7649 (29.6)	12 779 (27.9)	12 739 (25.2)	2100 (22.9)	1064 (21.3)	<.001
Past	1424 (18.3)	4889 (18.9)	8730 (19.1)	9835 (19.5)	1983 (21.6)	1045 (20.9)	
Current	3961 (50.8)	13 322 (51.5)	24 259 (53.0)	27 996 (55.4)	5097 (55.5)	2895 (57.9)	
Alcohol consumption							
Nondrinker	4105 (52.7)	11 870 (45.9)	17 766 (38.8)	19 160 (37.9)	3311 (36.1)	2111 (42.2)	<.001
<150 g/wk of ethanol	1505 (19.3)	5725 (22.1)	10 965 (24.0)	10 631 (21.0)	1636 (17.8)	837 (16.7)	
≥150 g/wk of ethanol	2187 (28.1)	8265 (32.0)	17 037 (37.2)	20 779 (41.1)	4233 (46.1)	2056 (41.1)	
BMI							
<18.5	396 (5.1)	1015 (3.9)	1542 (3.4)	1852 (3.7)	424 (4.6)	270 (5.4)	<.001
18.5 to <25.0	5119 (65.7)	17 232 (66.6)	31 878 (69.7)	35 609 (70.4)	6528 (71.1)	3427 (68.5)	
25.0 to <30.0	2093 (26.8)	6939 (26.8)	11 443 (25.0)	12 137 (24.0)	2077 (22.6)	1177 (23.5)	
≥30.0	189 (2.4)	674 (2.6)	905 (2.0)	972 (1.9)	151 (1.6)	130 (2.6)	
Physical activity^d^							
Low	3909 (50.1)	12 439 (48.1)	20 789 (45.4)	24 439 (48.3)	4440 (48.4)	2565 (51.3)	<.001
Intermediate	1124 (14.4)	4402 (17.0)	8650 (18.9)	8428 (16.7)	1433 (15.6)	624 (12.5)	
High	2764 (35.5)	9019 (34.9)	16 329 (35.7)	17 703 (35.0)	3307 (36.0)	1815 (36.3)	
Hypertension	2702 (34.7)	8081 (31.3)	13 041 (28.5)	15 877 (31.4)	3262 (35.5)	1948 (38.9)	<.001
Diabetes	558 (7.2)	1590 (6.2)	2577 (5.6)	2979 (5.9)	638 (7.0)	413 (8.3)	<.001

Abbreviations: BMI, body mass index (calculated as weight in kilograms divided by height in meters squared); NA, not applicable.

^a^ Data are presented as number (percentage) of men unless otherwise indicated.

^b^ The χ^2^ test was used for categorical variables and analysis of variance for continuous variables.

^c^ The percentage denotes the proportion of all men.

^d^ Physical activity was defined according to each cohort’s questionnaire. Low physical activity: once per week or <1 hour per week; intermediate physical activity: 1 to 4 days per week or 1 to 4 hours per week; high physical activity: almost daily or ≥5 hours per week.

**Table 2.** Baseline Characteristics for 178 542 East Asian Women According to Sleep Duration^a^

Characteristic	Sleep duration, h						*P* value^b^
	≤5	6	7	8	9	≥10	
No. of individuals^c^	13 564 (7.6)	38 340 (21.5)	60 319 (33.8)	53 109 (29.8)	8970 (5.0)	4240 (2.4)	NA
Age, mean (SD), y	58.1 (9.6)	55.3 (9.4)	54.0 (8.9)	55.3 (8.8)	57.5 (9.4)	58.7 (10.3)	<.001
Married	10 699 (78.9)	31 717 (82.7)	50 540 (83.8)	43 822 (82.5)	7105 (79.2)	3165 (74.7)	<.001
Smoking status							
Never	12 454 (91.8)	35 335 (92.2)	56 085 (93.0)	49 687 (93.6)	8335 (92.9)	3901 (92.0)	<.001
Past	227 (1.7)	601 (1.6)	844 (1.4)	703 (1.3)	129 (1.4)	90 (2.1)	
Current	883 (6.5)	2404 (6.3)	3390 (5.6)	2719 (5.1)	506 (5.6)	249 (5.9)	
Alcohol consumption							
Nondrinker	11 772 (86.8)	31 674 (82.6)	49 256 (81.7)	45 493 (85.7)	7854 (87.6)	3752 (88.5)	<.001
<150 g/wk of ethanol	1252 (9.2)	4753 (12.4)	7928 (13.1)	5291 (10.0)	813 (9.1)	342 (8.1)	
≥150 g/wk of ethanol	540 (4.0)	1913 (5.0)	3135 (5.2)	2325 (4.4)	303 (3.4)	146 (3.4)	
BMI							
<18.5	694 (5.1)	1605 (4.2)	2467 (4.1)	2144 (4.0)	414 (4.6)	203 (4.8)	<.001
18.5 to <25.0	8869 (65.4)	25 994 (67.8)	41 756 (69.2)	35 689 (67.2)	5749 (64.1)	2557 (60.3)	
25.0 to <30.0	3467 (25.6)	9293 (24.2)	14 335 (23.8)	13 391 (25.2)	2430 (27.1)	1220 (28.8)	
≥30.0	534 (3.9)	1448 (3.8)	1761 (2.9)	1885 (3.6)	377 (4.2)	260 (6.1)	
Physical activity^d^							
Low	6201 (45.7)	18 468 (48.2)	29 261 (48.5)	27 313 (51.4)	4483 (50.0)	2092 (49.3)	<.001
Intermediate	1964 (14.5)	5915 (15.4)	9412 (15.6)	7716 (14.5)	1293 (14.4)	510 (12.0)	
High	5399 (39.8)	13 957 (36.4)	21 646 (35.9)	18 080 (34.0)	3194 (35.6)	1638 (38.6)	
Hypertension	4351 (32.1)	10 550 (27.5)	15 860 (26.3)	15 602 (29.4)	3011 (33.6)	1450 (34.2)	<.001
Diabetes	697 (5.1)	1509 (3.9)	1999 (3.3)	2054 (3.9)	469 (5.2)	253 (6.0)	<.001
Menopause	10 222 (75.4)	25 334 (66.1)	37 666 (62.4)	35 318 (66.5)	6463 (72.1)	3037 (71.6)	<.001

Abbreviations: BMI, body mass index (calculated as weight in kilograms divided by height in meters squared); NA, not applicable.

^a^ Data are presented as number (percentage) of women unless otherwise indicated.

^b^ The χ^2^ test was used for categorical variables and analysis of variance for continuous variables.

^c^ The percentage denotes the proportion of all women.

^d^ Physical activity was defined according to each cohort’s questionnaire. Low physical activity: once per week or <1 hour per week; intermediate physical activity: 1 to 4 days per week or 1 to 4 hours per week; high physical activity: almost daily or ≥5 hours per week.

### All-Cause Mortality

**Figure 1.**
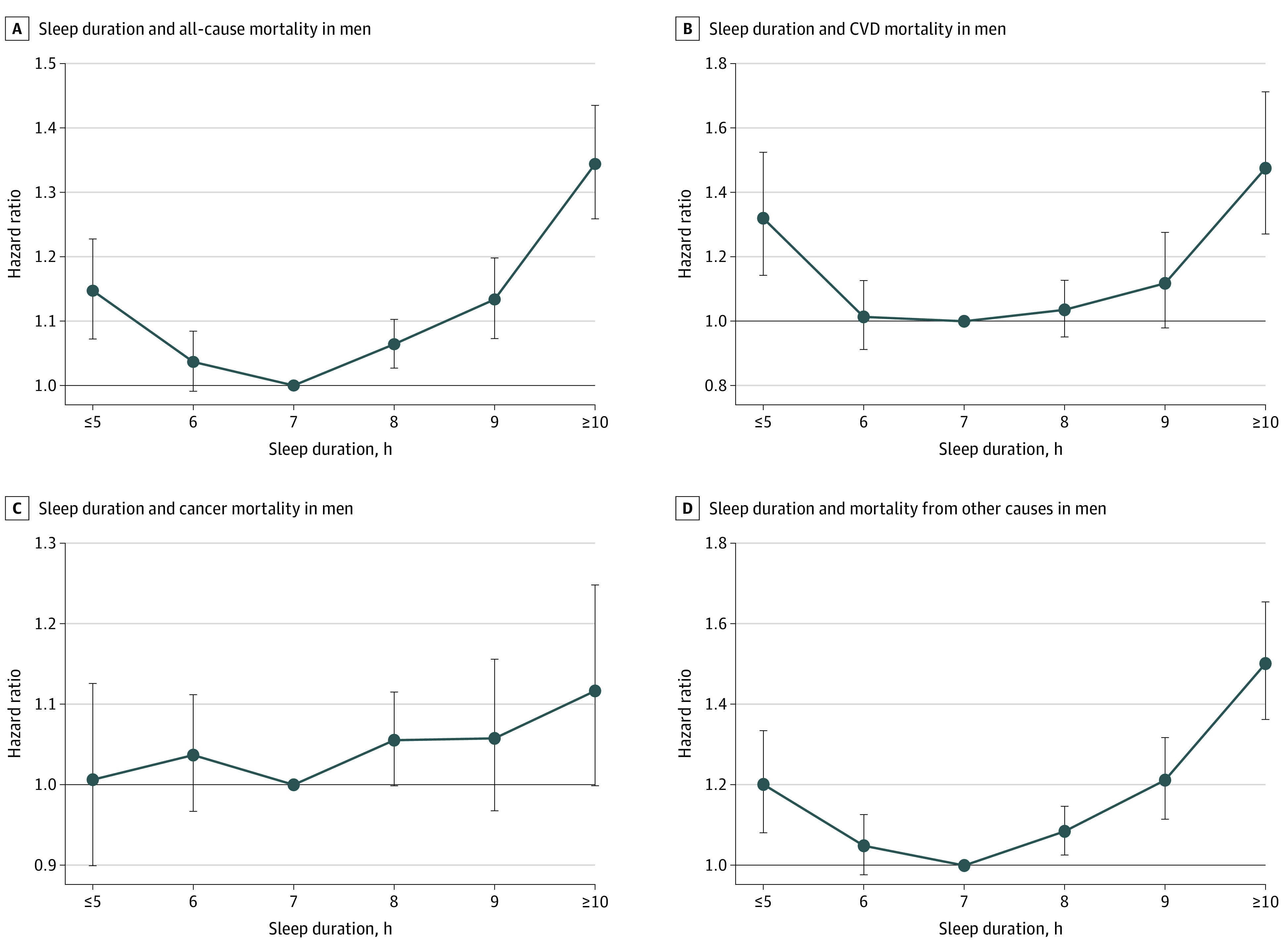
Association Between Sleep Duration and Mortality Risk in East Asian Men Hazard ratios and 95% CIs are shown for the association between sleep duration and mortality risk. The analyses were adjusted for age, marital status, study area (Japan Public Health Center–based studies only), body mass index, smoking, alcohol intake, physical activity, history of diabetes, and hypertension. Error bars indicate 95% CIs. CVD indicates cardiovascular disease.

**Figure 2.**
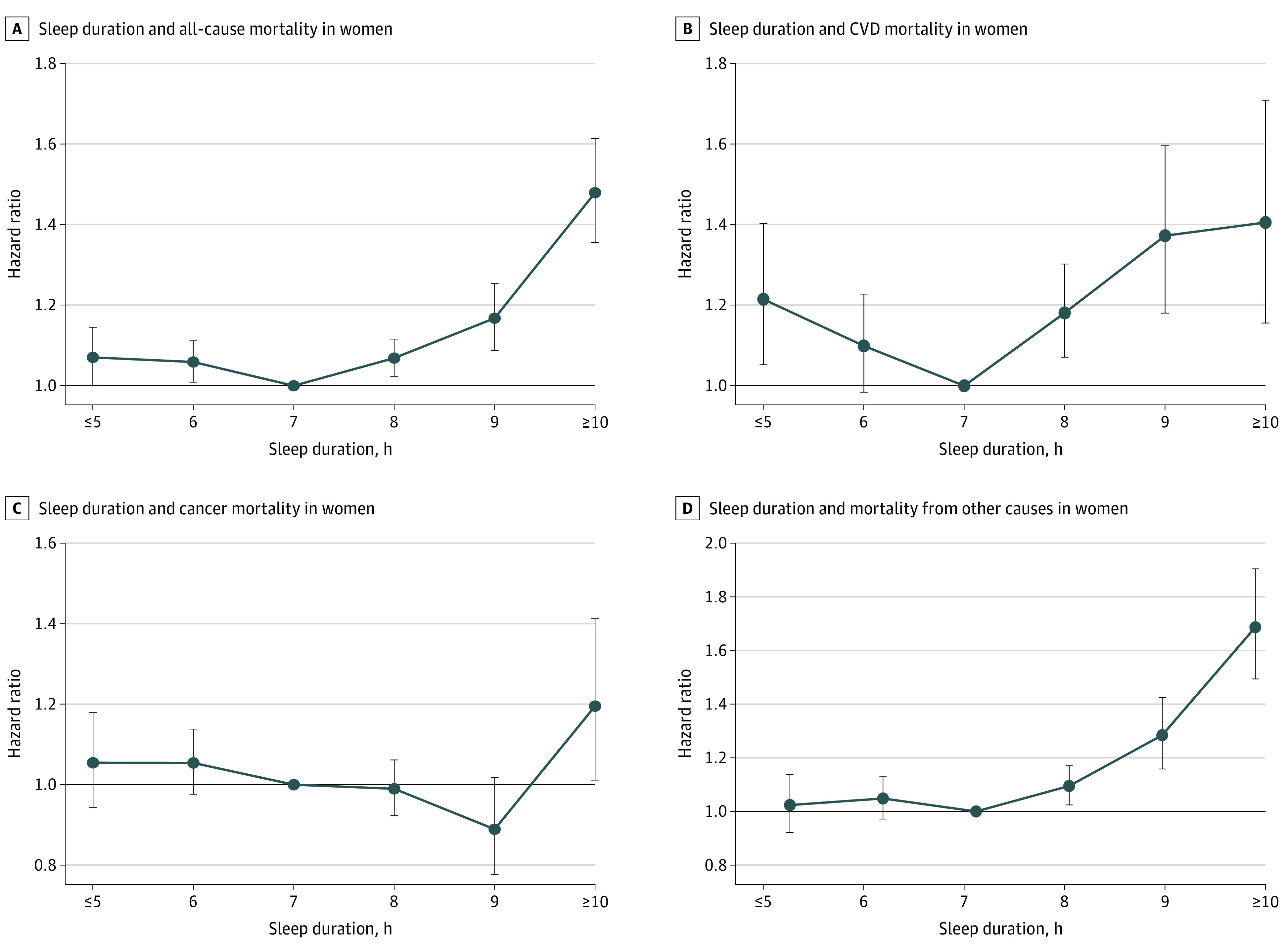
Association Between Sleep Duration and Mortality Risk in East Asian Women Hazard ratios and 95% CIs are shown for the association between sleep duration and mortality risk. The analyses were adjusted for age, marital status, study area (Japan Public Health Center–based studies only), body mass index, smoking, alcohol intake, physical activity, history of diabetes, hypertension, and menopausal status. Error bars indicate 95% CIs. CVD indicates cardiovascular disease.

A total of 19 419 deaths occurred among men and 13 768 among women during follow-up. The association between sleep duration and all-cause mortality was J-shaped for both men (Figure 1A; eTable 2 in the Supplement) and women (Figure 2A; eTable 3 in the Supplement). Sex was not a modifier of sleep duration for all-cause mortality (χ^2^_5_ = 8.64, *P* = .12). Compared with the referent 7 hours of sleep, the minimally and multivariable adjusted hazard ratios (HRs) were increased for all but 1 (6 hours) of the sleep duration categories for men and for all sleep duration categories for women. The strongest associations between sleep duration and mortality for both men (HR, 1.43, 95% CI, 1.34-1.53) and women (HR, 1.55, 95% CI, 1.42-1.70) were found for participants with the longest sleep duration (≥10 hours). Multivariable adjustment only slightly attenuated these associations (men: HR, 1.34, 95% CI, 1.26-1.44; women: HR, 1.48, 95% CI, 1.36-1.61). The results of age- and BMI-stratified analyses and their interaction with sleep duration in men and women are detailed in the eResults and eTables 4 through 7 in the Supplement.

### Cause-Specific Mortality

#### CVD Mortality

Sex was a statistically significant modifier of CVD mortality (χ^2^_5_ = 13.47, *P* = .02). In men, the association between sleep duration and CVD mortality was seen only with 5 hours or less, 9 hours, and 10 hours or more of sleep (Figure 1B; eTable 2 in the Supplement). Multivariable adjustment abrogated the association with 9 hours but only slightly attenuated the results for less than 5 hours and 10 hours or more. In women, the risk of CVD mortality was increased in all but 1 (6 hours) of the sleep duration categories in the minimally and multivariable adjusted models (Figure 2B; eTable 3 in the Supplement).

#### Cancer Mortality

Sex was a statistically significant modifier of the association between sleep duration and cancer mortality (χ^2^_5_ = 16.04, *P* = .007). In the minimally adjusted models for men, sleep durations of 8 hours, 9 hours, and 10 hours or more were associated with cancer mortality (Figure 1C; eTable 2 in the Supplement). Multivariable adjustment abrogated these associations. In women, a sleep duration of 10 hours or more was associated with cancer mortality in the minimally and multivariable adjusted models (Figure 2C; eTable 3 in the Supplement).

#### Mortality From Other Causes

Sex was a statistically significant modifier of the association between sleep duration and other-cause mortality (χ^2^_5_ = 12.79, *P* = .03). An increased risk of other-cause mortality was found for all but 1 of the sleep duration categories (6 hours) in men, with the strongest association observed for a sleep duration of 10 hours or more in the minimally and multivariable adjusted models (Figure 1D; eTable 2 in the Supplement). In women, the mortality risk from other causes was increased with sleep durations of 8 hours, 9 hours, and 10 hours or more in the minimally and multivariable adjusted models (Figure 2D; eTable 3 in the Supplement).

## Discussion

This cohort study was a pooled analysis and, to our knowledge, the largest study in Asia on the association between sleep duration and mortality outcomes. Multivariable-adjusted results suggest a clear association between sleep duration and all-cause mortality in both men and women. These overall associations support the results of sex-stratified analyses in several previous individual studies^13,14,15^ and meta-analyses.^2,5^ Our results further suggest a clear association between sleep duration and mortality from CVD and from causes other than CVD and cancer. Moreover, they offer novel insights into age as a modifier for the association between sleep duration and mortality risks among men.

An important reason to conduct analyses specifically in Asian populations is the increased likelihood of short sleep among Asians^16^ and stronger associations of long sleep duration with mortality in this racial group compared with other populations.^1,3^ In our study, there was a nadir of risk at 7 hours of sleep for associations with all-cause, CVD, and other-cause mortality in both men and women. However, 8 hours of sleep was the mode sleep duration among men and the second most common sleep duration among women. The results of the current study thus suggest that 35% of East Asian men and 29% of East Asian women whose sleep durations of 8 hours are in accord with current sleep duration recommendations of the US National Sleep Foundation^17^ may be at an increased risk of death from all causes and other causes (men and women) as well as CVD (women only).

The US National Sleep Foundation’s recommendations^18^ consider only age as a stratum and do not consider additional sex-specific differences. The possibility of sex-specific associations between sleep duration and all-cause mortality has been suggested in other studies.^6,8^ Previous studies are inconsistent in their findings of sex-specific associations and have reported higher risk of all-cause mortality with short sleep duration in men,^8,19,20^ short sleep duration in women,^21,22,23^ and long sleep duration in women.^24^ Despite reporting sex-specific results, few studies have included or investigated the necessary interaction terms that are required for the discussion of association modification by sex. In the current study, the interaction between sex and sleep duration was not statistically significant for all-cause mortality, which is in accord with a recent study from Korea.^25^ However, the interaction between sex and sleep duration in our study was significant for CVD, cancer, and other-cause mortality. Men had higher risks of CVD mortality than did women with sleep durations of 5 hours or less and 10 hours or more. Women, on the other hand, were at a statistically significantly increased risk of CVD mortality also at sleep durations of 8 hours and 9 hours, indicating that women, unlike men, may have a dose-dependent risk increase for sleep durations that exceed the referent 7 hours of sleep. On the basis of the data available, it is not possible to ascertain the underlying mechanism for the observed sex-specific differences. Notably, our findings of dose-dependent risk increases are concordant with a large meta-analysis,^26^ which unfortunately was limited in that it did not consider stratification by sex or race. Further investigation into plausible explanatory mechanisms is necessary.

In our study, only women with a sleep duration of 10 hours or more were at increased risk of cancer mortality. This finding and its magnitude agree with a meta-analysis^2^ that found that long but not short sleep duration was associated with cancer mortality only in women. Similarly, a more recent meta-analysis^27^ found that the risk of cancer mortality was increased only with long sleep duration and that the association was stronger in women and in Asian populations, albeit without any effect modification. Our study provides additional information of a possible threshold for a sleep duration of 10 hours or more, specifically in women. These sex-specific differences could be attributable to the association of sleep with cancer sites (eg, breast cancer). One possible reason for our discrepant findings compared with 2 large Japanese prospective studies,^28,29^ of which 1 study^29^ is included in the current analysis, is study size, indicating that large populations may be required to detect sex-specific associations between sleep duration and cancer mortality in East Asian populations. The absence of any association between sleep duration and cancer mortality in men in the multivariable model in the current study is explained entirely by the included covariates that abrogated the significant associations in the minimally adjusted model. Specific covariates for future studies to focus on and for which there were notable differences between the men and women of this study are smoking and alcohol consumption.

In the current study, age was a statistically significant modifier of the association between sleep duration and all-cause, cancer, and other-cause mortality in men only. Younger but not older men with sleep durations less than 7 hours were at a statistically significantly increased risk of all-cause mortality. In addition, men younger than 65 years had a stronger association with sleep durations that exceeded 7 hours than men older than 65 years. A previous study^9^ reported that increasing age results in a decreased risk of all-cause mortality associated with sleep duration. One finding that could offer a potential explanation for our results is that total sleep time in healthier populations is longer in men compared with women and decreases with age.^30^ Consequently, younger and healthier men may have a greater biological need for sleep that, on curtailment or extension, may result in adverse health outcomes. The term *biological need* would thus not equate to a need for more sleep but instead refer to a physiologically more stringent regulation compared with older men. The increased mortality risks among younger men in our study, as indicated by the highly significant interactions between age and sleep duration, were noted specifically for other-cause and cancer mortality. Given that these associations may differ depending on cancer type,^31^ future studies should aim to answer whether these findings can be attributed to any specific cancer types.

Mortality risks are more strongly affected by a low BMI in Asian populations.^11^ However, in the current study, BMI did not significantly modify the association between sleep duration and any of the mortality outcomes. These results, in particular the absence of any modification by BMI on CVD mortality, are surprising given the association between BMI and obstructive sleep apnea.^32^

The possible mechanisms of the associations between sleep duration and mortality are beyond the scope of the current study. One suggested mechanism pointing toward a causal pathway between short sleep and mortality is the association between short sleep and reduced leptin and increased ghrelin.^33^ This upregulation of appetite may explain the associations between short sleep and obesity^34^ and incident diabetes.^35^ In turn, further associations among obesity, diabetes, and mortality outcomes are well known. An in-depth review of the potential mechanisms that link short sleep duration and mortality has been conducted elsewhere.^36^ In contrast, the association between long sleep duration and mortality has been discussed in the context of residual confounding and comorbidities.^1^ Individuals with long sleep durations may already have an undiagnosed underlying disease process that may result in release of proinflammatory markers; both C-reactive protein and interleukin 6 are associated with long sleep duration.^37^

### Strengths and Limitations

This study has several strengths. First, it is the largest pooled analysis in Asia, and its size allowed us to estimate with a considerable degree of certainty the risk of death based on the habitual sleep durations of an East Asian population. Second, the results are probably generalizable across East Asian populations because the current study includes representative samples of adults from China, Japan, Singapore, and Korea. Third, analyses have been adjusted for important confounders of the association between sleep duration and mortality outcomes. Fourth, we had access to individual-level data, which avoids the limitations of reviews and meta-analyses. Moreover, meta-analyses are limited by the heterogeneous definition of *short* and *long* sleep durations. Consequently, this study purposefully avoided using such terms to describe our data and results. Instead, we have focused on quantifying risks in terms of specific sleep durations to contribute to future sleep recommendations and guidelines. Fifth, to minimize the effect of reverse causality, we focused on participants without CVD or cancer at baseline and excluded those who died in the first 5 years of follow-up. Despite such rigorous exclusions, we found significant associations between sleep durations and mortality outcomes.

This study also has some limitations. First, sleep duration was assessed using self-reported questionnaires, which may lead to an overestimation of sleep duration, in particular among those who sleep less.^38^ This approach may in turn underestimate the strength of the found associations between shorter sleep duration and the respective outcomes. Moreover, the sleep questionnaires were not standardized across the cohorts; some questionnaires requested information on nighttime sleep only, whereas others requested the combined duration of nighttime sleep and daytime naps. Although this may affect the interpretation of our results, more than 50% of participants provided information on their 24-hour habitual sleep duration. Second, sleep duration was measured only at 1 point, thereby not allowing us to consider changes in sleep duration over time. Third, this study excluded participants with a self-reported history of CVD and cancer at baseline, which may be subject to reporting bias and thus not capture all participants with the disease of interest. Moreover, in the context of the current analyses, individuals with prevalent diseases other than CVD or cancer may have remained in the final analysis. The inclusion of these participants may lead to an overestimation of our findings owing to a differential association of chronic diseases with sleep duration. Fourth, we need to consider the possibility of residual confounding because we did not have any information about sleep quality and other potentially relevant confounders (eg, depression and socioeconomic status). Fifth, causality cannot be inferred given that the results are derived from observational studies.

## Conclusions

The results of this study suggest that in an East Asian population, the mortality risks associated with sleep duration are modified by sex. In East Asian men, mortality risks are further modified by age. Future studies should investigate such modification toward specific mortality outcomes.

## Notes

Supplement. **eMethods.** Supplemental Methods **eResults.** Supplemental Results **eFigure.** Flowchart of Participant Inclusion and Exclusion **eTable 1.** Participating Cohort Characteristics **eTable 2.** HRs and CIs for Mortality According to Sleep Duration for Men **eTable 3.** HRs and CIs for Mortality According to Sleep Duration for Women **eTable 4.** HRs and CIs for Mortality According to Sleep Duration in Men Stratified by Age (Younger/Older Than 65 Years of Age) **eTable 5.** HRs and CIs for Mortality According to Sleep Duration in Women Stratified by Age (Younger/Older Than 65 Years of Age) **eTable 6.** HRs and CIs for Mortality for Men According to Sleep Duration Stratified by Body Mass Index (BMI) **eTable 7.** HRs and CIs for Mortality for Women According to Sleep Duration Stratified by Body Mass Index (BMI)
