# Supplementary material for: Association of Sleep Duration With All- and Major-Cause Mortality Among Adults in Japan, China, Singapore, and Korea
Source: JAMA Netw Open. 2021 Sep 3;4(9):e2122837. doi: 10.1001/jamanetworkopen.2021.22837 (PMC8417759; doi:10.1001/jamanetworkopen.2021.22837)
Supplement: Supplement. — eMethods. Supplemental Methods eResults. Supplemental Results eFigure. Flowchart of Participant Inclusion and Exclusion eTable 1. Participating Cohort Characteristics eTable 2. HRs and CIs for Mortality According to Sleep Duration for Men eTable 3. HRs and CIs for Mortality According to Sleep Duration for Women eTable 4. HRs and CIs for Mortality According to Sleep Duration in Men Stratified by Age (Younger/Older Than 65 Years of Age) eTable 5. HRs and CIs for Mortality According to Sleep Duration in Women Stratified by Age (Younger/Older Than 65 Years of Age) eTable 6. HRs and CIs for Mortality for Men According to Sleep Duration Stratified by Body Mass Index (BMI) eTable 7. HRs and CIs for Mortality for Women According to Sleep Duration Stratified by Body Mass Index (BMI) [file jamanetwopen-e2122837-s001.pdf]

Supplementary Online Content

Svensson T, Saito E, Svensson AK, et al. Association of sleep duration with all- and major-cause mortality among adults in Japan, China, Singapore, and Korea. *JAMA Netw Open*. 2021;4(9):e2122837. doi:10.1001/jamanetworkopen.2021.22837

eMethods. Supplemental Methods
eResults. Supplemental Results
eFigure. Flowchart of Participant Inclusion and Exclusion
eTable 1. Participating Cohort Characteristics
eTable 2. HRs and CIs for Mortality According to Sleep Duration for Men
eTable 3. HRs and CIs for Mortality According to Sleep Duration for Women
eTable 4. HRs and CIs for Mortality According to Sleep Duration in Men Stratified by Age (Younger/Older Than 65 Years of Age)
eTable 5. HRs and CIs for Mortality According to Sleep Duration in Women Stratified by Age (Younger/Older Than 65 Years of Age)
eTable 6. HRs and CIs for Mortality for Men According to Sleep Duration Stratified by Body Mass Index (BMI)
eTable 7. HRs and CIs for Mortality for Women According to Sleep Duration Stratified by Body Mass Index (BMI)

This supplementary material has been provided by the authors to give readers additional information about their work.

© 2021 Svensson T et al. *JAMA Network Open.*

eMethods. Supplemental Methods

Data harmonization

Data harmonization for the ACC is done centrally by the ACC Coordinating Center. Once a project proposal has been accepted by the ACC Executive Committee, the ACC Coordinating Center requests data from each participating cohort on a project-by-project basis. All cohorts relevant for a specific project (decided by the availability of exposure and outcome measures) receive an invitation to participate. The number of cohorts participating in each project varies depending on the research question and availability of data. The ACC Coordinating Center cleans and pools the collected data and provides principal investigators with access to the finalized dataset. Data can be analysed either remotely using a virtual private network, or on-site at the National Cancer Center in Tokyo. The access to ACC data requires that the principal investigator agrees to and signs a data use agreement form.

Main exposure

The main exposure of the present study was self-reported sleep duration. Three of the cohorts (the Korean Multi-Center Cancer Cohort Study (KMCC), the Singapore Chinese Health Study (SCHS), and the Takayama Study) asked about sleep duration as a categorical variable, and the remaining six cohorts (Japan Public Health Center-based prospective (JPHC) Study 1 and JPHC Study 2, Miyagi Cohort Study, Ohsaki National Health Insurance Cohort Study, Shanghai Men's Health Study (SMHS), and SWHS) asked about sleep duration as a continuous variable.

The participants of each cohort were asked about their sleep duration as follows: **Japan Public Health Center-based prospective Study 1** and **2**: "How many hours do you usually sleep?"; **Miyagi Cohort Study**: "How many hours on average do you sleep per day?"; **Ohsaki National Health Insurance Cohort Study**: "How many hours on average do you sleep per day?"; **Takayama Study**: "How long did you sleep on average during the past year? (including naptime)?"; **Shanghai Men's Health Study**: "In the past year, how many hours did you sleep each day? (including sleeping at day and night, but not including the time you woke up between two periods of sleep)?" **Shanghai Women's Health Study**: "In the past 2 years, how many hours did you sleep each day (including sleeping during the day and night, but not including time if you woke up between two periods of sleep); **Singapore Chinese Health Study**: "On the average, during the last year, how many hours did you sleep in a day, including naps?"; **Korean Multi-center Cancer Cohort Study**: "During the past year, on average per day, how many hours of sleep have you had (including nap time)?"

Outcome ascertainment and follow-up

The outcomes of the present study were mortality from all causes (ICD-9: 001-999, ICD-10: A00-G99, I00-N99, Q00-T98, V00-Y99), CVD (ICD-9: 410-414 and 430-438, ICD-10: I20-I25 and I60-I69), cancer (ICD-9: 140-208, ICD-10: C00-C96), and other causes (mortality other than CVD or cancer).

Covariates

Age (continuous) was obtained at the start of follow-up; study area was relevant for the JPHC study; information on marital status (yes/no), body mass index (BMI; <18.5 kg/m^2, 18.5-24.9 kg/m^2, 25-29.9 kg/m^2, and ≥30 kg/m^2), smoking status (never smoker, past smoker, or current

© 2021 Svensson T et al. *JAMA Network Open.*

smoker), alcohol consumption (non-drinker/past drinker, current drinker <150g ethanol/week, and current drinker ≥150g ethanol/week), physical activity (low: once per week or < 1 hour per week; intermediate: 1-4 days per week or 1-4 hours per week; high: almost daily or ≥ 5 hours per week) according to each cohort's questionnaire, history of diabetes, history of hypertension (prevalent hypertension, systolic blood pressure ≥ 140, or diastolic blood pressure ≥90), and menopausal status (for women only) were all based on self-reported information. For the SWHS, information on menopausal status and prevalent hypertension was derived from both the baseline questionnaire and the third follow-up survey.

eResults. Supplemental Results

All-cause mortality

Age was a statistically significant effect modifier for all-cause mortality among men (p<0.001) but not women (p=0.62). When stratifying analyses by age, younger men were at an increased risk of all-cause mortality with all sleep duration categories compared to the referent 7 hours (eTable 4). Among older men, there was an increased risk only with sleep durations of 9 hours, and ≥10 hours in both the minimally- and multivariable adjusted model.

BMI was not an effect modifier for all-cause mortality in men (p=0.33) or women (p=0.67). Analyses stratified by BMI are shown in eTables 6 and 7.

CVD mortality

In sex-stratified analyses, neither age (men: p=0.44; women: p=0.34; eTables 4 and 5) nor BMI (men: p=0.87; women: p=0.95; eTables 6 and 7) were effect modifiers for the association between sleep duration and CVD mortality.

Cancer mortality

Among men, age (p<0.001) but not BMI (p=0.88) was a statistically significant effect modifier of the association between sleep duration and cancer mortality. In age-stratified analyses, younger men had an increased risk of cancer mortality with sleep durations of ≤5 hours, 8 hours, 9 hours, and ≥10 hours in both the minimally- and multivariable adjusted models. Sleep duration was unrelated to risk of cancer mortality among older men.
In women, neither age (p=0.38) nor BMI (p=0.64) were effect modifiers of the association between sleep duration and cancer mortality.

Other-cause mortality

In men, age (p=0.002) but not BMI (p=0.27) was a statistically significant effect modifier of the association between sleep duration and mortality from other causes. Age-stratified analyses revealed increased risks for younger men with sleep durations of ≤5 hours, 8 hours, 9 hours, and ≥10 hours in both models. Older men were at an increased risk of other-cause mortality only with sleep durations of 9 hours, and ≥10 hours in both models.

Neither age (p=0.17) nor BMI (p=0.29) were effect modifiers for other-cause mortality in women.

© 2021 Svensson T et al. *JAMA Network Open.*

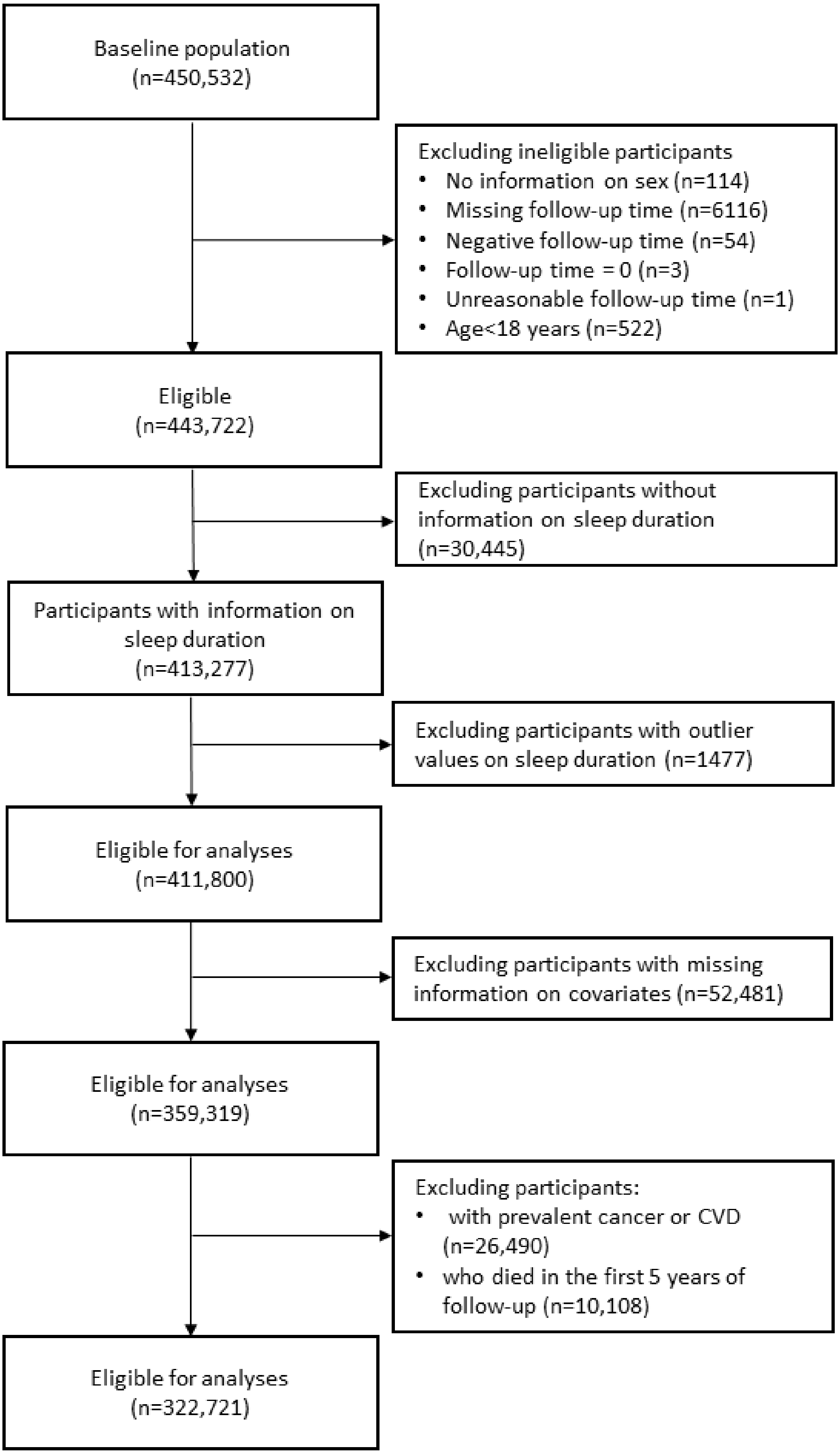

eFigure 1. Flowchart of participant inclusion and exclusion

© 2021 Svensson T et al. *JAMA Network Open.*

eTable 1. Participating cohort characteristics*

	Cohort																	
	JPHC I		JPHC II		Miyagi		Ohsaki		Takayama		SMHS		SWHS		SCHS		KMCC	
Follow-up time, mean (SD) years	21.4 (3.5)		18.3 (3.2)		16.4 (3.2)		11.5 (3.7)		14.3 (3.2)		9.2 (1.0)		8.3 (0.8)		12.0 (2.1)		14.3 (4.2)	
Participants	N	%	N	%	N	%	N	%	N	%	N	%	N	%	N	%	N	%
Total	38954	100	47904	100	31623	100	29896	100	20430	100	34453	100	58089	100	55519	100	5853	100
Men	18706	48.0	22184	46.3	17423	55.1	15907	53.2	9577	46.9	34453	100	0	0	24044	43.3	1885	32.2
Women	20248	52.0	25720	53.7	14200	44.9	13989	46.8	10853	53.1	0	0	58089	100	31475	56.7	3968	67.8
Deaths	5544	14.2	8508	17.8	3005	9.5	3266	10.9	2630	12.9	1395	4.1	2048	3.5	6033	10.9	758	13.0
Proportionate mortality																		
CVD	870	15.7	1383	16.3	543	18.1	623	19.1	436	16.6	249	17.9	421	20.6	1635	27.1	133	17.6
Cancer	2339	42.2	3257	38.3	1378	45.9	1163	35.6	856	32.6	688	49.3	837	40.9	2148	35.6	230	30.3
Other	2335	42.1	3868	45.5	1084	36.1	1480	45.3	1338	50.9	458	32.8	790	38.6	2250	37.3	395	52.1
Sleep duration (Men)																		
≤5 hours	578	3.1	830	3.7	503	2.9	334	2.1	266	2.8	3181	9.2	-	-	1923	8.0	182	9.7
6 hours	2570	13.7	3466	15.6	2508	14.4	1670	10.5	1555	16.2	8044	23.4	-	-	5622	23.4	425	22.6
7 hours	6170	33.0	7005	31.6	6275	36.0	4566	28.7	3896	40.7	9299	27.0	-	-	8042	33.5	515	27.3
8 hours	7758	41.5	8335	37.6	6673	38.3	6714	42.2	2982	31.1	10632	30.9	-	-	6927	28.8	549	29.1
9 hours	1157	6.2	1695	7.6	1042	6.0	1650	10.4	685	7.2	1870	5.4	-	-	967	4.0	114	6.1
≥10 hours	473	2.5	853	3.9	422	2.4	973	6.1	193	2.0	1427	4.1	-	-	563	2.3	100	5.3
Sleep duration (Women)																		
≤5 hours	1075	5.3	1203	4.7	588	4.1	603	4.3	419	3.9	-	-	5925	10.2	3220	10.2	531	13.4
6 hours	4059	20.1	5164	20.1	2900	20.4	2320	16.6	2677	24.7	-	-	12923	22.3	7419	23.6	878	22.1
7 hours	7754	38.3	9403	36.6	5865	41.3	4565	32.6	4506	41.5	-	-	16722	28.8	10440	33.2	1064	26.8
8 hours	6428	31.8	8140	31.7	4134	29.1	4890	35.0	2550	23.5	-	-	17568	30.2	8341	26.5	1058	26.7
9 hours	695	3.4	1312	5.1	534	3.8	1032	7.4	493	4.5	-	-	3195	5.5	1416	4.5	293	7.4
≥10 hours	237	1.2	498	1.9	179	1.3	579	4.1	208	1.9	-	-	1756	3.0	639	2.0	144	3.6

*Cohort characteristics are shown according to the final study population (i.e., individuals who are complete cases without any prevalent cardiovascular disease (CVD) or cancer at baseline, and who did not die within the first five years of follow-up).

Abbreviations: JPHC=Japan Public Health Center; SMHS=Shanghai Men’s Health Study; SWHS=Shanghai Women’s Health Study; SCHS=Singapore Chinese Health Study; KMCC= Korean Multi-Center Cancer Cohort.

© 2021 Svensson T et al. *JAMA Network Open.*

eTable 2 HRs and CIs for mortality according to sleep duration for men

		**Men (n=144,179)**					
		Sleep duration (h)					
		≤5	6	7	8	9	≥10
Person-years		96,216	345,256	657,426	729,309	129,293	64,789
**Cause of death**							
**All-cause**							
No. (Events)		7797 (1029)	25,860 (2978)	45,768 (5395)	50,570 (7198)	9180 (1700)	5004 (1119)
Model 1, HR (95% CI)		**1.16*** (1.08, 1.24)**	1.03 (0.99, 1.08)	Reference	**1.09*** (1.05, 1.13)**	**1.18*** (1.11, 1.24)**	**1.43*** (1.34, 1.53)**
Model 2, HR (95% CI)		**1.15*** (1.07, 1.23)**	1.04 (0.99, 1.08)	Reference	**1.06*** (1.03, 1.10)**	**1.13*** (1.07, 1.20)**	**1.34*** (1.26, 1.44)**
**CVD**							
No. (Events)		7797 (238)	25,860 (549)	45,768 (967)	50,570 (1231)	9180 (294)	5004 (220)
Model 1, HR (95% CI)		**1.35*** (1.17, 1.56)**	1.01 (0.91, 1.12)	Reference	1.06 (0.97, 1.15)	**1.17* (1.03, 1.34)**	**1.59*** (1.37, 1.84)**
Model 2, HR (95% CI)		**1.32*** (1.14, 1.52)**	1.01 (0.91, 1.13)	Reference	1.04 (0.95, 1.13)	1.12 (0.98, 1.28)	**1.48*** (1.27, 1.71)**
**Cancer**							
No. (Events)		7797 (363)	25,860 (1237)	45,768 (2258)	50,570 (2962)	9180 (639)	5004 (371)
Model 1, HR (95% CI)		1.01 (0.90, 1.13)	1.03 (0.96, 1.11)	Reference	**1.08** (1.02, 1.14)**	**1.10* (1.00, 1.20)**	**1.19** (1.06, 1.33)**
Model 2, HR (95% CI)		1.01 (0.90, 1.13)	1.04 (0.97, 1.11)	Reference	1.06 (1.00, 1.12)	1.06 (0.97, 1.16)	1.12 (1.00, 1.25)
**Other cause**							
No. (Events)		7797 (428)	25,860 (1192)	45,768 (2170)	50,570 (3005)	9180 (767)	5004 (528)
Model 1, HR (95% CI)		**1.22*** (1.10, 1.36)**	1.04 (0.97, 1.12)	Reference	**1.11*** (1.05, 1.17)**	**1.26*** (1.16, 1.36)**	**1.60*** (1.45, 1.76)**
Model 2, HR (95% CI)		**1.20*** (1.08, 1.33)**	1.05 (0.98, 1.13)	Reference	**1.08** (1.03, 1.15)**	**1.21*** (1.11, 1.32)**	**1.50*** (1.36, 1.65)**

Model 1 was adjusted for age, marital status, and study area (JPHC Study only)
Model 2 was further adjusted for body mass index, smoking, alcohol intake, physical activity, history of diabetes, and hypertension
**p*<0.05, ****p*<0.001 vs reference

© 2021 Svensson T et al. *JAMA Network Open.*

eTable 3 HRs and CIs for mortality according to sleep duration for women

		Women (n=178,542)					
		Sleep duration (h)					
		≤5	6	7	8	9	≥10
Person-years		162,656	507,564	842,907	714,776	114,102	50,032
**Cause of death**							
**All-cause**							
No. (Events)		13,564 (1110)	38,340 (2776)	60,319 (4083)	53,109 (4248)	8970 (947)	4240 (604)
Model 1, HR (95% CI)		**1.10** (1.03, 1.18)**	**1.07** (1.02, 1.13)**	Reference	**1.08*** (1.03, 1.13)**	**1.21*** (1.13, 1.30)**	**1.55*** (1.42, 1.70)**
Model 2, HR (95% CI)		**1.07* (1.00, 1.15)**	**1.06* (1.01, 1.11)**	Reference	**1.07** (1.02, 1.12)**	**1.17*** (1.09, 1.25)**	**1.48*** (1.36, 1.61)**
**CVD**							
No. (Events)		13,564 (261)	38,340 (552)	60,319 (750)	53,109 (885)	8970 (225)	4240 (121)
Model 1, HR (95% CI)		**1.24** (1.08, 1.43)**	1.12 (1.00, 1.25)	Reference	**1.20*** (1.09, 1.32)**	**1.45*** (1.25, 1.68)**	**1.49*** (1.22, 1.81)**
Model 2, HR (95% CI)		**1.21** (1.05, 1.40)**	1.10 (0.98, 1.23)	Reference	**1.18*** (1.07, 1.30)**	**1.37*** (1.18, 1.60)**	**1.41*** (1.16, 1.71)**
**Cancer**							
No. (Events)		13,564 (394)	38,340 (1092)	60,319 (1667)	53,109 (1513)	8970 (246)	4240 (154)
Model 1, HR (95% CI)		1.07 (0.96, 1.20)	1.06 (0.99, 1.15)	Reference	0.99 (0.93, 1.06)	0.90 (0.79, 1.03)	**1.23* (1.04, 1.45)**
Model 2, HR (95% CI)		1.05 (0.94, 1.18)	1.05 (0.98, 1.14)	Reference	0.99 (0.92, 1.06)	0.89 (0.78, 1.02)	**1.20* (1.01, 1.41)**
**Other cause**							
No. (Events)		13,564 (455)	38,340 (1132)	60,319 (1666)	53,109 (1850)	8970 (476)	4240 (329)
Model 1, HR (95% CI)		1.06 (0.95, 1.17)	1.06 (0.99, 1.15)	Reference	**1.11** (1.04, 1.18)**	**1.35*** (1.22, 1.49)**	**1.77*** (1.57, 2.00)**
Model 2, HR (95% CI)		1.02 (0.92, 1.14)	1.05 (0.97, 1.13)	Reference	**1.09** (1.02, 1.17)**	**1.28*** (1.16, 1.42)**	**1.69*** (1.49, 1.90)**

Model 1 was adjusted for age, marital status, and study area (JPHC Study only)
Model 2 was further adjusted for body mass index, smoking, alcohol intake, physical activity, history of diabetes, hypertension, and menopausal status
**p*<0.05, ***p*<0.01, ****p*<0.001 vs reference

© 2021 Svensson T et al. *JAMA Network Open.*

eTable 4 HRs and CIs for mortality according to sleep duration in men stratified by age (younger/older than 65 years of age)

		**Age<65 (n=124,867)**							**Age≥65 (n=19,312)**					
		Sleep duration (h)							Sleep duration (h)					
		≤5	6	7	8	9	≥10		≤5	6	7	8	9	≥10
Person-years		80,693	307,679	601,510	653,162	108,617	50,644		15,523	37,577	55,916	76,147	20,676	14,145
**Cause of death**														
**All-cause**														
No. (Events)		6339 (628)	22,424 (2038)	40,884 (3930)	44,070 (5120)	7402 (1044)	3748 (612)		1458 (401)	3436 (940)	4884 (1465)	6500 (2078)	1778 (656)	1256 (507)
Model 1, HR (95% CI)		**1.34*** (1.23, 1.46)**	**1.07* (1.01, 1.12)**	Reference	**1.20*** (1.15, 1.25)**	**1.47*** (1.38, 1.58)**	**1.98*** (1.82, 2.15)**		1.09 (0.98, 1.22)	1.03 (0.95, 1.12)	Reference	1.03 (0.96, 1.10)	**1.20*** (1.09, 1.31)**	**1.42*** (1.28, 1.57)**
Model 2, HR (95% CI)		**1.30*** (1.20, 1.42)**	**1.06* (1.01, 1.12)**	Reference	**1.16*** (1.11, 1.21)**	**1.37*** (1.28, 1.47)**	**1.78*** (1.64, 1.94)**		1.07 (0.95, 1.19)	1.03 (0.94, 1.11)	Reference	1.01 (0.94, 1.08)	**1.17*** (1.07, 1.28)**	**1.36*** (1.23, 1.51)**
**CVD**														
No. (Events)		6339 (135)	22,424 (367)	40,884 (685)	44,070 (821)	7402 (177)	3748 (113)		1458 (103)	3436 (182)	4884 (282)	6500 (410)	1778 (117)	1256 (107)
Model 1, HR (95% CI)		**1.55*** (1.28, 1.86)**	1.07 (0.94, 1.21)	Reference	**1.12* (1.01, 1.24)**	**1.49*** (1.26, 1.76)**	**2.14*** (1.75, 2.61)**		**1.33* (1.06, 1.67)**	0.99 (0.82, 1.19)	Reference	1.09 (0.94, 1.27)	1.18 (0.95, 1.47)	**1.64*** (1.31, 2.06)**
Model 2, HR (95% CI)		**1.47*** (1.22, 1.77)**	1.06 (0.93, 1.20)	Reference	1.08 (0.97, 1.19)	**1.35*** (1.15, 1.60)**	**1.84*** (1.50, 2.24)**		**1.30* (1.03, 1.63)**	0.98 (0.81, 1.18)	Reference	1.08 (0.93, 1.26)	1.16 (0.93, 1.44)	**1.60*** (1.27, 2.00)**
**Cancer**														
No. (Events)		6339 (244)	22,424 (900)	40,884 (1731)	44,070 (2246)	7402 (445)	3748 (226)		1458 (119)	3436 (337)	4884 (527)	6500 (716)	1778 (194)	1256 (145)
Model 1, HR (95% CI)		**1.16* (1.02, 1.33)**	1.06 (0.98, 1.15)	Reference	**1.19*** (1.12, 1.27)**	**1.43*** (1.29, 1.59)**	**1.65*** (1.43, 1.89)**		0.90 (0.74, 1.10)	1.02 (0.89, 1.17)	Reference	0.97 (0.87, 1.09)	0.98 (0.83, 1.15)	1.10 (0.91, 1.32)
Model 2, HR (95% CI)		**1.15* (1.00, 1.31)**	1.06 (0.98, 1.15)	Reference	**1.16*** (1.09, 1.24)**	**1.35*** (1.22, 1.50)**	**1.52*** (1.32, 1.75)**		0.92 (0.75, 1.11)	1.03 (0.89, 1.18)	Reference	0.95 (0.85, 1.06)	0.95 (0.80, 1.12)	1.04 (0.87, 1.26)
**Other cause**														
No. (Events)		6339 (249)	22,424 (771)	40,884 (1514)	44,070 (2053)	7402 (422)	3748 (273)		1458 (179)	3436 (421)	4884 (656)	6500 (952)	1778 (345)	1256 (255)
Model 1, HR (95% CI)		**1.45*** (1.27, 1.66)**	1.07 (0.98, 1.17)	Reference	**1.23*** (1.16, 1.32)**	**1.52*** (1.36, 1.69)**	**2.29*** (2.01, 2.61)**		1.13 (0.96, 1.34)	1.06 (0.93, 1.19)	Reference	1.04 (0.94, 1.15)	**1.38*** (1.21, 1.57)**	**1.57*** (1.36, 1.82)**
Model 2, HR (95% CI)		**1.40*** (1.22, 1.60)**	1.07 (0.98, 1.17)	Reference	**1.20*** (1.12, 1.28)**	**1.41*** (1.27, 1.57)**	**2.05*** (1.80, 2.34)**		1.08 (0.91, 1.28)	1.05 (0.92, 1.18)	Reference	1.02 (0.93, 1.13)	**1.34*** (1.17, 1.53)**	**1.52*** (1.31, 1.76)**

Model 1 was adjusted for marital status, and study area (JPHC Study only)

Model 2 was further adjusted for body mass index, smoking, alcohol intake, physical activity, history of diabetes, and hypertension

**p*<0.05, ***p*<0.01, ****p*<0.001 vs reference

© 2021 Svensson T et al. *JAMA Network Open.*

eTable 5 HRs and CIs for mortality according to sleep duration in women stratified by age (younger/older than 65 years of age)

	**Age<65 (n=147,786)**						**Age≥65 (n=30,756)**					
	Sleep duration (h)						Sleep duration (h)					
	≤5	6	7	8	9	≥10	≤5	6	7	8	9	≥10
Person-years	125,159	435,493	753,027	617,505	89,585	36,317	37,497	72,072	89,880	97,271	24,517	13,714
**Cause of death**												
**All-cause**												
No.<br>(Events)	9806<br>(515)	31,363<br>(1624)	52,156<br>(2740)	44,616<br>(2602)	6844<br>(444)	3001<br>(232)	3758<br>(595)	6977<br>(1152)	8163<br>(1343)	8493<br>(1646)	2126<br>(503)	1239<br>(372)
Model 1, HR<br>(95% CI)	**1.23*****<br>**(1.12 1.35)**	1.06<br>(1.00, 1.13)	Reference	**1.19*****<br>**(1.12, 1.25)**	**1.44*****<br>**(1.31, 1.60)**	**1.96*****<br>**(1.71, 2.24)**	**1.13***<br>**(1.03, 1.25)**	**1.11****<br>**(1.03, 1.20)**	Reference	**1.13*****<br>**(1.05, 1.21)**	**1.34*****<br>**(1.21, 1.49)**	**1.84*****<br>**(1.64, 2.06)**
Model 2, HR<br>(95% CI)	**1.14****<br>**(1.03 1.25)**	1.04<br>(0.98, 1.11)	Reference	**1.12*****<br>**(1.07, 1.18)**	**1.28*****<br>**(1.16, 1.41)**	**1.70*****<br>**(1.48, 1.94)**	**1.11***<br>**(1.00, 1.22)**	**1.10***<br>**(1.02, 1.19)**	Reference	**1.11****<br>**(1.03, 1.20)**	**1.28*****<br>**(1.16, 1.42)**	**1.76*****<br>**(1.57, 1.98)**
**CVD**												
No.<br>(Events)	9806<br>(91)	31,363<br>(272)	52,156<br>(457)	44,616<br>(483)	6844<br>(93)	3001<br>(45)	3758<br>(170)	6977<br>(280)	8163<br>(293)	8493<br>(402)	2126<br>(132)	1239<br>(76)
Model 1, HR<br>(95% CI)	**1.27***<br>**(1.01, 1.59)**	1.06<br>(0.91, 1.23)	Reference	**1.33*****<br>**(1.17, 1.51)**	**1.80*****<br>**(1.44, 2.25)**	**2.26*****<br>**(1.66, 3.07)**	**1.42*****<br>**(1.17, 1.72)**	**1.21***<br>**(1.03, 1.43)**	Reference	**1.29*****<br>**(1.11, 1.50)**	**1.66*****<br>**(1.35, 2.04)**	**1.76*****<br>**(1.36, 2.26)**
Model 2, HR<br>(95% CI)	1.13<br>(0.90, 1.41)	1.04<br>(0.89, 1.21)	Reference	**1.22****<br>**(1.08, 1.39)**	**1.51*****<br>**(1.21, 1.89)**	**1.80*****<br>**(1.33, 2.46)**	**1.39*****<br>**(1.15, 1.68)**	**1.20***<br>**(1.02, 1.41)**	Reference	**1.27****<br>**(1.09, 1.48)**	**1.56*****<br>**(1.27, 1.92)**	**1.65*****<br>**(1.28, 2.13)**
**Cancer**												
No.<br>(Events)	9806<br>(233)	31,363<br>(745)	52,156<br>(1290)	44,616<br>(1091)	6844<br>(141)	3001<br>(85)	3758<br>(161)	6977<br>(347)	8163<br>(377)	8493<br>(422)	2126<br>(105)	1239<br>(69)
Model 1, HR<br>(95% CI)	**1.17***<br>**(1.02, 1.35)**	1.03<br>(0.94, 1.12)	Reference	1.05<br>(0.97, 1.14)	0.97<br>(0.82, 1.16)	**1.50*****<br>**(1.20, 1.87)**	1.05<br>(0.88, 1.27)	**1.17***<br>**(1.01, 1.36)**	Reference	1.03<br>(0.90, 1.19)	1.03<br>(0.83, 1.28)	1.27<br>(0.98, 1.65)
Model 2, HR<br>(95% CI)	1.11<br>(0.97, 1.28)	1.02<br>(0.93, 1.11)	Reference	1.02<br>(0.94, 1.11)	0.91<br>(0.76, 1.08)	**1.39****<br>**(1.12, 1.74)**	1.05<br>(0.87, 1.27)	**1.17***<br>**(1.01, 1.35)**	Reference	1.03<br>(0.89, 1.18)	1.00<br>(0.81, 1.25)	1.25<br>(0.96, 1.61)
**Other cause**												
No.<br>(Events)	9806<br>(191)	31,363<br>(607)	52,156<br>(993)	44,616<br>(1028)	6844<br>(210)	3001<br>(102)	3758<br>(264)	6977<br>(525)	8163<br>(673)	8493<br>(822)	2126<br>(266)	1239<br>(227)
Model 1, HR<br>(95% CI)	**1.29*****<br>**(1.10, 1.51)**	1.10<br>(1.01, 1.22)	Reference	**1.29*****<br>**(1.19, 1.41)**	**1.89*****<br>**(1.63, 2.20)**	**2.43*****<br>**(1.98, 2.99)**	1.05<br>(0.91, 1.21)	1.04<br>(0.92, 1.16)	Reference	**1.11***<br>**(1.00, 1.23)**	**1.37*****<br>**(1.19, 1.58)**	**2.15*****<br>**(1.85, 2.50)**
Model 2, HR<br>(95% CI)	1.17<br>(1.00, 1.36)	1.08<br>(0.98, 1.20)	Reference	**1.20*****<br>**(1.10, 1.31)**	**1.62*****<br>**(1.39, 1.88)**	**2.00*****<br>**(1.63, 2.45)**	1.01<br>(0.87, 1.17)	1.02<br>(0.91, 1.15)	Reference	1.09<br>(0.98, 1.21)	**1.31*****<br>**(1.13, 1.51)**	**2.06*****<br>**(1.76, 2.39)**

Model 1 was adjusted for marital status, and study area (JPHC Study only)
Model 2 was further adjusted for body mass index, smoking, alcohol intake, physical activity, history of diabetes, hypertension, and menopause
**p*<0.05, ***p*<0.01, ****p*<0.001 vs reference

© 2021 Svensson T et al. *JAMA Network Open.*

eTable 6 HRs and CIs for mortality for men according to sleep duration stratified by body mass index (BMI)

	**BMI<25 kg/m^2 (n=105,292)**						**BMI≥25 kg/m^2 (n=38,887)**					
	Sleep duration (h)						Sleep duration (h)					
	≤5	6	7	8	9	≥10	≤5	6	7	8	9	≥10
Person-years	67,128	242,560	480,866	541,919	98,183	48,035	29,089	102,697	176,560	187,390	31,110	16,754
**Cause of death**												
**All-cause**												
No. (Events)	5515 (801)	18,247 (2228)	33,420 (4138)	37,461 (5625)	6952 (1367)	3697 (890)	2282 (228)	7613 (750)	12,348 (1257)	13,109 (1573)	2228 (333)	1307 (229)
Model 1, HR (95% CI)	**1.21*** (1.12, 1.30)**	1.03 (0.98, 1.09)	Reference	**1.09*** (1.05, 1.14)**	**1.19*** (1.12, 1.27)**	**1.46*** (1.35, 1.57)**	1.03 (0.89, 1.19)	1.04 (0.95, 1.14)	Reference	1.07 (1.00, 1.16)	1.11 (0.99, 1.26)	**1.34*** (1.16, 1.54)**
Model 2, HR (95% CI)	**1.20*** (1.11, 1.30)**	1.04 (0.99, 1.10)	Reference	**1.07** (1.02, 1.11)**	**1.15*** (1.08, 1.22)**	**1.37*** (1.28, 1.48)**	1.03 (0.89, 1.19)	1.03 (0.94, 1.13)	Reference	1.07 (0.99, 1.15)	1.10 (0.98, 1.25)	**1.30*** (1.12, 1.49)**
**CVD**												
No. (Events)	5515 (181)	18,247 (400)	33,420 (724)	37,461 (944)	6952 (234)	3697 (172)	2282 (57)	7613 (149)	12,348 (243)	13,109 (287)	2228 (60)	1307 (48)
Model 1, HR (95% CI)	**1.41*** (1.19, 1.66)**	1.01 (0.90, 1.15)	Reference	1.06 (0.97, 1.17)	**1.19* (1.03, 1.39)**	**1.62*** (1.37, 1.92)**	1.20 (0.90, 1.61)	1.01 (0.83, 1.25)	Reference	1.05 (0.88, 1.24)	1.08 (0.81, 1.43)	**1.44* (1.05, 1.97)**
Model 2, HR (95% CI)	**1.38*** (1.17, 1.63)**	1.02 (0.90, 1.15)	Reference	1.04 (0.94, 1.14)	1.14 (0.98, 1.32)	**1.52*** (1.28, 1.80)**	1.18 (0.88, 1.57)	1.01 (0.82, 1.24)	Reference	1.03 (0.87, 1.23)	1.05 (0.79, 1.40)	1.36 (0.99, 1.86)
**Cancer**												
No. (Events)	5515 (272)	18,247 (921)	33,420 (1719)	37,461 (2286)	6952 (517)	3697 (291)	2282 (91)	7613 (316)	12,348 (539)	13,109 (676)	2228 (122)	1307 (80)
Model 1, HR (95% CI)	1.02 (0.90, 1.16)	1.04 (0.96, 1.12)	Reference	**1.08* (1.02, 1.15)**	**1.14* (1.03, 1.25)**	**1.21** (1.07, 1.38)**	0.98 (0.78, 1.22)	1.03 (0.89, 1.18)	Reference	1.07 (0.95, 1.20)	0.95 (0.78, 1.16)	1.10 (0.87, 1.39)
Model 2, HR (95% CI)	1.02 (0.90, 1.17)	1.05 (0.97, 1.13)	Reference	1.05 (0.99, 1.12)	1.09 (0.99, 1.20)	1.13 (1.00, 1.28)	0.98 (0.78, 1.23)	1.02 (0.89, 1.17)	Reference	1.06 (0.95, 1.19)	0.95 (0.78, 1.16)	1.08 (0.85, 1.36)
**Other cause**												
No. (Events)	5515 (348)	18,247 (907)	33,420 (1695)	37,461 (2395)	6952 (616)	3697 (427)	2282 (80)	7613 (285)	12,348 (475)	13,109 (610)	2228 (151)	1307 (101)
Model 1, HR (95% CI)	**1.30*** (1.15, 1.46)**	1.04 (0.96, 1.13)	Reference	**1.11*** (1.04, 1.18)**	**1.24*** (1.13, 1.36)**	**1.61*** (1.45, 1.79)**	1.00 (0.79, 1.27)	1.08 (0.93, 1.25)	Reference	1.09 (0.97, 1.23)	**1.31** (1.09, 1.58)**	**1.55*** (1.24, 1.93)**
Model 2, HR (95% CI)	**1.28*** (1.14, 1.44)**	1.05 (0.96, 1.13)	Reference	**1.09** (1.02, 1.16)**	**1.21*** (1.10, 1.33)**	**1.54*** (1.38, 1.71)**	1.00 (0.79, 1.27)	1.07 (0.92, 1.24)	Reference	1.09 (0.96, 1.23)	**1.30** (1.08, 1.57)**	**1.50*** (1.21, 1.87)**

Model 1 was adjusted for age, marital status, and study area (JPHC Study only)
Model 2 was further adjusted for smoking, alcohol intake, physical activity, history of diabetes, and hypertension
**p*<0.05, ***p*<0.01, ****p*<0.001 vs reference

© 2021 Svensson T et al. *JAMA Network Open.*

eTable 7 HRs and CIs for mortality for women according to sleep duration stratified by body mass index (BMI)

	**BMI<25 kg/m^2 (n=128,141)**						**BMI≥25 kg/m^2 (n=50,401)**					
	Sleep duration (h)						Sleep duration (h)					
	≤5	6	7	8	9	≥10	≤5	6	7	8	9	≥10
Person-years	114,992	368,230	623,034	513,049	78,636	32,591	47,664	139,334	219,873	201,727	35,466	17,441
**Cause of death**												
**All-cause**												
No. (Events)	9563 (764)	27,599 (1916)	44,223 (2887)	37,833 (2966)	6163 (636)	2760 (402)	4001 (346)	10,741 (860)	16,096 (1196)	15,276 (1282)	2807 (311)	1480 (202)
Model 1, HR (95% CI)	**1.10* (1.01, 1.19)**	**1.06* (1.00, 1.13)**	Reference	**1.08** (1.02, 1.14)**	**1.19*** (1.09, 1.30)**	**1.57*** (1.41, 1.75)**	1.11 (0.98, 1.20)	**1.10* (1.00, 1.19)**	Reference	1.08 (1.00, 1.17)	**1.25*** (1.10, 1.42)**	**1.50*** (1.29, 1.75)**
Model 2, HR (95% CI)	1.08 (0.99, 1.17)	1.05 (0.99, 1.11)	Reference	**1.07* (1.02, 1.13)**	**1.15*** (1.06, 1.26)**	**1.53*** (1.37, 1.70)**	1.09 (0.96, 1.23)	1.09 (1.00, 1.19)	Reference	1.07 (0.99, 1.16)	**1.21** (1.07, 1.37)**	**1.42*** (1.22, 1.65)**
**CVD**												
No. (Events)	9563 (181)	27,599 (375)	44,223 (510)	37,833 (590)	6163 (149)	2760 (79)	4001 (80)	10,741 (177)	16,096 (240)	15,276 (295)	2807 (76)	1480 (42)
Model 1, HR (95% CI)	**1.30** (1.09, 1.54)**	1.13 (0.99, 1.29)	Reference	**1.19** (1.06, 1.34)**	**1.47*** (1.22, 1.77)**	**1.55*** (1.22, 1.98)**	1.14 (0.88, 1.48)	1.08 (0.89, 1.32)	Reference	**1.22* (1.03, 1.45)**	**1.40* (1.08, 1.82)**	1.30 (0.93, 1.82)
Model 2, HR (95% CI)	**1.28** (1.08, 1.52)**	1.12 (0.98, 1.28)	Reference	**1.17** (1.04, 1.32)**	**1.40*** (1.16, 1.68)**	**1.51*** (1.19, 1.93)**	1.11 (0.86, 1.43)	1.08 (0.89, 1.31)	Reference	**1.20* (1.01, 1.43)**	**1.33* (1.03, 1.73)**	1.22 (0.87, 1.71)
**Cancer**												
No. (Events)	9563 (276)	27,599 (745)	44,223 (1190)	37,833 (1035)	6163 (155)	2760 (99)	4001 (118)	10,741 (347)	16,096 (477)	15,276 (478)	2807 (91)	1480 (55)
Model 1, HR (95% CI)	1.09 (0.95, 1.24)	1.03 (0.94, 1.13)	Reference	0.97 (0.89, 1.06)	**0.84* (0.71, 0.99)**	1.22 (0.99, 1.50)	1.04 (0.85, 1.28)	1.14 (0.99, 1.31)	Reference	1.04 (0.92, 1.18)	1.03 (0.82, 1.29)	1.24 (0.93, 1.64)
Model 2, HR (95% CI)	1.07 (0.93, 1.22)	1.02 (0.93, 1.12)	Reference	0.97 (0.89, 1.05)	**0.83* (0.70, 0.98)**	1.19 (0.97, 1.47)	1.03 (0.84, 1.27)	1.13 (0.99, 1.30)	Reference	1.04 (0.91, 1.18)	1.03 (0.82, 1.29)	1.22 (0.92, 1.62)
**Other cause**												
No. (Events)	9563 (307)	27,599 (796)	44,223 (1187)	37,833 (1341)	6163 (332)	2760 (224)	4001 (148)	10,741 (336)	16,096 (479)	15,276 (509)	2807 (144)	1480 (105)
Model 1, HR (95% CI)	1.02 (0.89, 1.16)	1.06 (0.97, 1.16)	Reference	**1.13** (1.05, 1.22)**	**1.34*** (1.19, 1.52)**	**1.77*** (1.53, 2.05)**	1.16 (0.96, 1.40)	1.07 (0.93, 1.23)	Reference	1.05 (0.93, 1.19)	**1.35** (1.12, 1.63)**	**1.75*** (1.41, 2.17)**
Model 2, HR (95% CI)	1.00 (0.88, 1.14)	1.05 (0.96, 1.15)	Reference	**1.12** (1.04, 1.21)**	**1.29*** (1.14, 1.46)**	**1.73*** (1.49, 2.00)**	1.14 (0.94, 1.37)	1.06 (0.92, 1.22)	Reference	1.04 (0.91, 1.17)	**1.28* (1.06, 1.55)**	**1.61*** (1.29, 1.99)**

Model 1 was adjusted for age, marital status, and study area (JPHC Study only)

Model 2 was further adjusted for smoking, alcohol intake, physical activity, history of diabetes, hypertension, and menopausal status

*p<0.05, **p<0.01, ***p<0.001 vs reference

© 2021 Svensson T et al. *JAMA Network Open.*
